# Prevalence of *Ascaris lumbricoides* in contaminated faecal samples of children residing in urban areas of Lahore, Pakistan

**DOI:** 10.1038/s41598-020-78743-y

**Published:** 2020-12-11

**Authors:** Shahida Azhar Ali, Sadaf Niaz, Liliana Aguilar-Marcelino, Wajid Ali, Majid Ali, Adil Khan, Sohail Amir, Nasreen, Abdullah D. Alanazi, Raquel Cossio-Bayugar, Itzel Amaro-Estrada

**Affiliations:** 1 Department of Zoology, University of the Punjab, Quaid-i-Azam Campus, Lahore, Pakistan; 2 Department of Zoology, Abdul Wali Khan University Mardan, Mardan, Pakistan; 3 Center for Integrated Mountain Research, University of the Punjab, Lahore, Pakistan; 4 Centro Nacional de Investigación Disciplinaria en Salud Animal e Inocuidad, INIFAP, Km 11 Carretera Federal Cuernavaca-Cuautla, No. 8534, Col. Progreso, CP 62550 Jiutepec, Morelos Mexico; 5 Hayat Abad Medical Complex, Peshawar, Pakistan; 6 Department of Biological Sciences, Faculty of Science and Humanities, Shaqra University, P.O. Box 1040, Ad-Dawadimi, 11911 Saudi Arabia

**Keywords:** Zoology, Diseases, Medical research

## Abstract

Ascariasis is a common public health problem of preschool and primary school children in developing countries like Pakistan. The aim of the present study was to determine the prevalence and pattern of *Ascaris lumbericoides* (*A. lumbricoides*) infection among children residing in urban areas of Lahore, to provide information on ascariasis to promote awareness and prevention programs between the participants specially on the months or season of higher prevalence. To investigate the prevalence of *Ascaris Lumbricoides* in the contaminated faecal samples of children residing in urban areas of Lahore, a study was conducted from November 2010 to October 2012 and we collected 3600 stratified faecal samples from six urban study areas. Overall 32/3600 (0.88%) prevalence of fecal samples was found positive for eggs of *Ascaris lumbricoides*. Area wise highest presence positivity 1.67% was observed in Allama Iqbal Town followed by 1.17% in Samanabad, 1.00% in Wapda Town, 1.00% in Gulberg, 0.50% in Cantt, and the lowest 0.00% in Valencia Town respectively (p < 0.001) The highest month wise positivity prevalence 3/300 (3.33%) (p < 0.001) was observed in the month of September that gradually declined up to 0/300 (0.00%) in the month of March. The results reveal that urban areas of Lahore are susceptible to *Ascaris Lubricoides* infection and the highest prevalence were observed autumn on the month of September.

## Introduction

About 2 billion people in the world are infected with at least one species of Soil Transmitted Helminths (STH) i.e., one billion due to *A. lumbricoides* and 4 billion are at risk^1^. Globally about 1.5 billion people are affected by *Ascaris lumbricoides* (*A. lumbricoides*) infection. Children are susceptible to infestation with environmental and socio-economic status which has influence on child health, as risk factors^2^.

*Ascaris lumbricoides* (*A. lumbricoides*) is a Soil Transmitted Helminth (STH) commonly distributed in tropical and sub-tropical areas, is a common nematode infecting human with increased prevalence (%) due to poor sanitary conditions. About 4 billion people are at risk, 613 million are specifically school-age children^3^.

Studies revealed that poor sociodemographic and socioeconomic status of the children are important factors for the presence of high prevalence of STH. The presence of STH is higher in rural areas than urban areas due to poor infrastructural facilities and improper sewage system^4^. Inadequate water supply, contact with contaminated soil, walking bare footed, do not wash hands before eating and after defecation in early childhood when they are incautious for self-hygiene, eating raw vegetables and low Socio-Economic Status (SES) enhanced the prevalence to get ascariasis^5^. The golden period for betterment in good physical and mental developmental health of children is in their early childhood, provided by the parents and society^6^.

Adult worm lives in small and large intestine^7,8^ of man and its eggs passed out via faeces^9^ of infected person to soil and contaminate it^10^. Eggs in soil are transmitted to water, vegetables, food, seats of commodes, handles of doors and in air by the insect’s wings and and legs, like house flies and cockroaches. Fertilized unsegmented eggs release with faeces of infected person, passed eggs are not infective, at 22–23 °C and moisture in soil enhances development, it is viable for 3 years under these suitable conditions in soil, ponds and sewage water. Healthy people become infected when ingest or inhale the infective larva, once in the body the larvae moves towards liver actively, resides in lungs where it grows into an adult within 5–10 days. Adult female lays about 200,000 eggs/day those are shed outside via faeces into soil, again the cycle is repeated^9^. Ascariasis is frequently promoted in domestic conditions as its eggs persist in household dust. Human is the significant hosts of STHs hence transmitted by fecal–oral-route.

*A. lumbricoides* has been reported as cause of larva migrants in humans and is generally assumed to be the most worldwide prevalent. It also caused stress in infected children with hypereosinophila along with Loeffler’s syndrome when larva of *A. lumbricoides* migrated from lungs^11^. In case of heavy infection causes intestinal obstruction (IO), intussusception, volvulus, intestinal necrosis and volvulus^2^.

Helminthic infections often lead to iron deficiency anaemia (IDA)^12^, protein energy malnutrition, stunting (a measure of chronic under nutrition), wasting (a measure of acute under nutrition), listlessness and abdominal pain and may negatively affect class performance of schoolchildren^13^.

STH presence is responsible for anaemia in infected children^14^ of age 1–12 years^13^ that leads to loss of appetite, depletion of absorption of micro-nutrients, loss of weight, irritability in respiratory system and cough when worms are present in lungs, vomiting, colic, diarrhea, obstruction of intestine, when they reside in duodenum/intestine, cognitive deficiency and stunting growth i.e. (40.4%—55.7%). If no medication, ascariasis reoccur and intestinal blockage with abdominal pain, wheezing like asthma, cough, short breathing or permanent fever are the secondary signs^15^.

When number of adult worms is abundant, they may come out of orifices like nose, mouth, vagina, anus or ears solitarily or in bunches form. The toxins release by adult worms causes fever, oedema on face, conjunctivitis, rashes, paraplegia, meningitis, irritation in upper respiratory tract, malnutrition and weight loss^16^.

Many surveys have been conducted in different cities of Pakistan to observe the prevalence ratios of *A. lumbricoides* in faecal samples of children, yet the research was done in limited areas, in minimum number, with very small size of data and without the observation of SES. Similarly, the data is in scattered form. Therefore, the aim of this study was to find out the prevalence (%) of infection in naturally infected children along with observing the personal hygiene and SES data. In Pakistan seasonal and climatic variations due to global changes have marked effects on ascariasis and it was observed that prevalence of *A. lumbricoides* was nearly equally distributed, both in faecal and soil samples of urban areas.

## Material and methods

### Ethical approval

The protocol for the study was approved by the institutional (Quaid-e-Azam University of the Punjab, Lahore, Pakistan and the Department of Zoology) ethics committee. Written approval was obtained from the respected parents of the subjects and M.S of the Shalamar Hospital, Lahore. The approval was on the agreement that patient anonymity must be maintained, good laboratory facility must be ensured. All work was performed according to the international guidelines for human experimentation in biomedical research. All children that were infected were treated.

### Collection samples

A total of 3600 faecal samples were collected between November 2010 to October 2012 from six different urban areas of Lahore, Pumjab, Pakistan (Allama Iqbal Town, Wapda Town, Valencia Town, Cantt, Gulberg and Samanabad). A random sampling approach was applied to collect faecal samples from 25 children/ month for two years form each urban area (n = 6). The participant children age range was between 5 to 10 years old. For the seasonal analyses the year was apportioned into 4 seasons with the following breakup winter (November- February), spring (March–April), summer (May–August) and autumn (September–October). The prevalence of nematodal infections was recorded in relation to area and socio-economic status of human beings. The month wise and seasonal prevalence was determined by the formula as described by Thrusfield^17^.

### Collection and faecal sample processing

The samples from school children were collected through school administration and the samples from unregistered children in schools through door-to-door visits with help of their parents. Explanation on how to collect sample were given and Screw caped plastic vials clearly labeled with name, sex, date and place were provided to school management and parents (door to door visit). The faecal samples were collected and immediately transferred to the parasitology laboratory, Department of Zoology, University of the Punjab, Lahore for examination. For the presence of eggs of STHs the samples were examined on the same day by direct microscopic technique and by sedimentation and flotation method^18^. The samples that were not processed on the same day were preserved in 10% formalin to prevent the eggs development and hatching. In addition, during studies the seasonal prevalence was also recorded.

### Direct microscopic examination

A small amount of fecal sample was mixed with saline solution (0.9%) in a petri dish and stirred it with glass rod to mix it thoroughly. Few drops of this homogeneous mixture were taken and were placed on glass slide, covered with cover glass. From each sample three slides were prepared following the above procedure. Helminth’s eggs were observed under microscope (10 × 10 and 10 × 40). Eggs were identified on the basis of morphology^19,20^ and prevalence of infection was monthly recorded i.e. area wise, and season wise.

### Sedimentation

In 5 ml of a 5% acetic acid solution one gram of faeces was suspended by shaking. The suspension was kept for one minute to settle and then filtered through a tea sieve into a centrifuge tube. An equal amount of ether was added, mixed and shaked vigorously and centrifuged for one minute at 1,500 rpm. The sediment containing eggs was formed in the centrifuge tube with above it the acid layer and the ether layer, between the ether and the acid layer there was layer of dirt that was removed from the tube by means of an applicator. The supernatant (ether, acid and dirt) was decanted all at once only sediment remained and that was diluted with water, mixed homogeneously, a few drops of that mixture was placed on glass slide^18^ and examined under microscope (10 × 10 and 10 × 40)^19^.

### Flotation technique

One gram of faecale sample was added to 10 ml of 4% NaCl solution and mixed thoroughly. The suspension was poured into a test tube. Additional 4% NaCl solution was added to test tube till overflow. A cover slip was placed on the upper surface of the tube, left for 10 to 15 min, then removed the cover slip vertically and examined under microscope (10 ×)^18^.

The prevalence of the infection/disease was recorded following the modified formula described by Thursfield^17^.

$${\text{Prevalence}}\;\left( \% \right) = \frac{{{\text{No}}.\;{\text{of}}\;{\text{infected}}\;{\text{individuals}}\;{\text{at}}\;{\text{particular}}\;{\text{point}}\;{\text{intime}}}}{{{\text{No}}\;{\text{of}}\;{\text{total}}\;{\text{individuals}}\;{\text{at}}\;{\text{particular}}\;{\text{point}}\;{\text{intime}}}} \times 100$$

### Statistical analysis

Data was analyzed statistically by using computer software, Microsoft SPSS (Statistical Products and Service Solution) (version 14.0) 10.0. < 0.05 was considered significant.

### Statistics for faecal samples

Prevalence values were calculated by dividing the number of positive samples by total number of samples analyzed, Z test ANOVA (Analysis of Variance) was applied by following Steel and Torri (1981)^21^, proportions were compared by calculating confidence intervals, variables were rated 1 (positive), 2 (negative). Binary logistic regression was set up with pre- determined significance level of 0.05 (p < 0.05).

## Results

**Figure 1:**
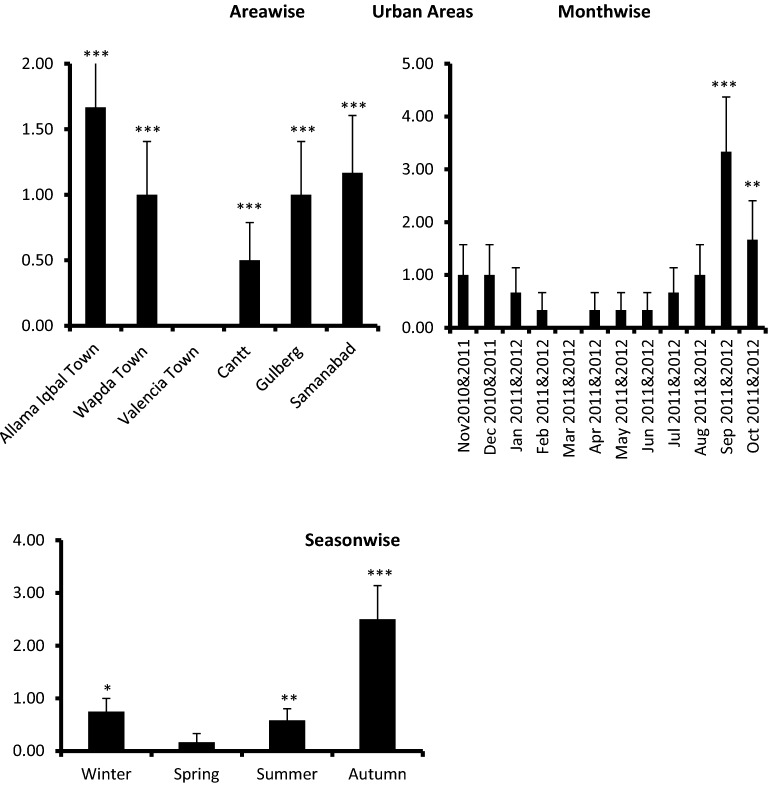
Presence (%) of *Ascaris lumbericoides’s* eggs in soil samples of urban areas of Lahore (**a**) area wise, (**b**) month wise, (**c**) and seasonwise from Nov 2010- Oct 2012. Z-test * = P < 0.05, ** = P < 0.01, ***P < .001.

For epidemiological survey, 3600 coprological samples of children (3600/area/2 years) were observed in 6 urban areas (Allama Iqbal Town, Wapda Town, Valencia Town, Cantt, Gulberg and Samanabad) of Lahore, Punjab, Pakistan, from November 2010 to October 2012, out of which 32/3600 (0.88%) the overall prevalence was found (Table 1; Fig. 1).

**Table 1:** Overall area wise, monthwise and seasonwise presence (%) of *Ascaris lumbricoides*’s eggs in soil samples of urban areas of Lahore, Punjab, Pakistan from November 2010—October 2012.

Factors	Total n = 3600		Infested	Presence (% ± S. E)
	Urban areas	Observed		
Areas	Allama Iqbal Town	(n = 3600 /year)	10/600	1.67 ± 0.52
	Wapda Town		6/600	1.00 ± 0.41***
	Valencia Town		0/600	0.00 ± 0.00***
	Cantt		3/600	0.50 ± 0.29***
	Gulberg		6/600	1.00 ± 0.41***
	Samanabad		7/600	1.17 ± 0.44***
Total			32/3600	0.88 ± 0.08***
Time (months)	Nov2010&2011	(n = 3600/month/year)	3/300	1.00 ± 0.57
	Dec 2010&2011		3/300	1.00 ± 0.57
	Jan 2011&2012		2/300	0.67 ± 0.57
	Feb 2011&2012		1/300	0.33 ± 0.33
	Mar 2011&2012		0/300	0.00 ± 0.00
	Apr 2011&2012		1/300	0.33 ± 0.33
	May 2011&012		1/300	0.33 ± 0.33
	Jun 2011&2012		1/300	0.33 ± 0.33
	Jul 2011&2012		2/300	0.67 ± 0.57
	Aug 2011&012		3/300	1.00 ± 0.57
	Sep 2011&2012		10/300	3.33 ± 1.04***
	Oct 2011&2012		5/300	1.67 ± 0.74**
Total			32/3600	0.88 ± 0.08***

*P < .05, **P < .01, ***P < .001 Z-test.

### Overall prevalence

An overall prevalence 32/3600 (0.88%) (p < 0.001) of ascariasis was found in urban of Lahore, Punjab from November 2010 to October 2012 (Table 1; Fig. 1).

### Area wise prevalence (%)

Area wise faecal prevalence (%) of naturally infected children in six urban areas of Lahore, Punjab province indicated the highest infection rate was Area wise highest presence positivity 10/600 (1.67%) was observed in Allama Iqbal Town, followed by 7/600 (1.17%) in Samanabad, 6/600 (1.00%) in Wapda Town, 6/600 (1.00%) in Gulberg, 3/600 (0.50%) in Cantt, 0.00% and the lowest in Valencia Town 0/600 (0.00%) respectively (p < 0.001) (Table 1; Fig. 1).

### Month wise prevalence (%)

After analyzing month wise data, the highest month-wise positivity prevalence 10/300 (3.33%) was observed in the month of September that gradually declined up to 0.00% in the month of March (Table 1; Fig. 1). No significant difference was observed between prevalence (%) of the month February, July and August. Whereas November (p < 0.001), September (p < 0.001) and October (p < 0.01) showed significant difference with respect to March (Table 1). When statistical analysis (Z-test) was applied on month wise data, prevalence (%) of March was the lowest in urban areas, so March was compared with other months.

### Season wise prevalence (%)

The highest prevalence in all urban areas of Lahore, Punjab was observed during autumn 15/600 (2.5%), followed by summer 7/1200 (0.58%), winter 9/1200 (0.75%), and lowest in spring 1/600 (0.17%) of urban (Fig. 1). Statistically prevalence was significantly higher in autumn (p < 0.001), summer (p < 0.01) and in winter (p < 0.05) as compared to spring (Table 1; Fig. 1).

## Discussion

Parasitic infestation and diseases are the public health problem in developing countries like Pakistan especially in areas with low Socio-Economic Status (SES), poor parental education, occupation, geographical status of localities, prevailing variable climatic conditions, poor personal hygiene, inadequate health care education and prevailed low sanitary conditions. The results of these findings revealed overall presence percentage 0.88% of *A. lumbricoides* in the faecal samples of naturally infected children indicated that they become risk for the human population. That faecal material not only contaminate the soil but also *A. lumbricoides* eggs stick to the vegetables i.e., carrots, spinach, salads, reddish, coriander, mint sugar cane etc. and other herbs grown in the nearby fields. The results of these findings revealed overall presence percentage 5.53% of *A. lumbricoides* in the soil samples less than 6.3% in Uganda^22^, 48. 8% in Brazil^23^, 54.1% Nigeria^24^, due to variation in lab technique. Similarly, it was observed the SES conditions in Pakistan are better than the mentioned countries. Therefore, both in eggs and larval forms *A. lumbricoides* can survive and ascariasis can be transmitted from one person to another due to gregarious behavior of children when living in clustering of family members with poor hygienic conditions, improper disposal of faeces and urbanization. These observations are supported by the findings of Pullan and Brooker^25^.

The use of night soil as fertilizers by the farmers increased the presence rate of infestation of ascariasis in soil, the children who played there barefooted and 90% had the habit of geophagia, thumb sucking, and nail biting as observed during this study. Insects like *Musca domestica* (housefly), cockroaches and the pet pups or stray dogs who sat in those contaminated places might have had *A. lumbeicoides* eggs stick to their furs. When children came in contact with them, they might ingest the eggs as they were found. They had not the habit to wash their hands before taking meal and after defecation, besides the younger children did not care to eat the fallen food on contaminated soil like bread, biscuits, banana. These findings are supported by the observations of Tilahun et al.^4^*.*

Therefore, children should be provided with better SES in early childhood with healthy environmental conditions, because SES has marked effects not only on health, cognitive status, and socio emotional outcome of children but also produces stress on both children and parents. In Pakistan, seasonal and climatic variations due to global changes have been observed from collected meteorological data. It was observed that prevalence of *A. lumbricoides* was nearly equally distributed in soil samples of urban areas. It was concluded that eggs of *A. lumbricoides* survive in soil for several months without any break, even in extremely hot and arid conditions and its larva resides in alimentary canal of human and restricted its development there for several months safely. Therefore, both eggs and larval forms *A. lumricoides* can survive and ascariasis can be transmitted from one person to another due to gregarious behavior of children when living in clustering of family members with poor hygienic conditions, improper disposal of faeces and urbanization. These observations are supported by the findings of Pullan and Brooker^25^.

Rain and air were also the media for transmission of *A. lumbeicoide*s eggs from one place to another helped to pollute the environment and had promoted ascariasis in the children of age 3–13 years old that was supported by the results of Nishiura et al.^26^.

Increased rate of ascariasis presence (%) was in those children who had poor socioeconomic status, poor personal hygiene, poor supply of drinking water, low grade sanitary conditions especially poor sewage system and lived-in endemic areas with clustering of family members, supported by the studies in Nigeria^24^; Muzaffarabad district^27^ and in Kashmir^28^, Pakistan. Presence of pups/stray dogs enhanced the presence as they acted as disseminators and transmitters of *A. lumbeicoides*’s eggs^29^ especially in stray dogs.

The prevalence rate in urban areas especially in Allama Iqbal town, Valancia and Wapada Town was due to the reason that these areas were under construction, nomads and daily wagers were residing there for earning and had the habit to defecate in open places. They were living in clustering family pattern and unaware about self-hygiene. The seeded faecal material not only contaminated the soil but also eggs stick to the vegetables i.e., carrots, spinach, salads, reddish, coriander, mint sugar cane etc., and other herbs grown in the nearby fields. Toilets had poor construction as under construction buildings had no proper sewage system. Similarly, the water supply was through the single tap or hand pump, installed there. They used to keep pail of water there and not only used for bathing but also wash utensils there, as toilets mostly deprived of door, just a cloth curtain hanged there, pets had easy access to toilet and wander here and there put mouth in pail of water and in order to avoid heat loved to sit there. These observations are consistent with Esfandairi et al.^30^. These unhygienic conditions were promoting infestation in soil and infection in human due to gregarious behavior of children. Farmers used the night soil as fertilizers also increased the prevalence rate of infestation of ascariasis in the children who played there barefooted and 90% had the habit of geophagia, sucking thumbs and nail biting as observed during present study.

Children had the habit to play with their pet pups or stray dogs who sit in those contaminated places might have had *A. lumbricoides*’s eggs stick to their furs. When children came in contact with them, they might ingest the eggs as they were found had the habit to not wash their hands before taking meal and after defecation, the younger children don’t care to eat the fallen food on contaminated soil like bread, biscuits, banana with their dirty hands, as careless about their self-hygiene. In this way aggravated contamination in the present study is consistent with observation of Steinmann et al.^31^. The younger’s feeder’s nipples and pacifiers were observed being covered with flies when present on khats (wooden rope beds) in open areas and were frequently used by them, even mother did not care about it, so insects also promoted transmission of eggs from one place to other. Especially parents were unaware about the importance of hygiene and biology of parasite. They were of the notion that they were made of mud and worms were the natural phenomenon, helpful in health of children, if they reside in their body either in the form of ectoparasites or endoparasites. The present study shows consistent trend in this regard with the observation of Anuar et al.^32^. Level of contamination of soil was aggravated during rainy seasons, as water contributes contamination of soils and transmission of eggs from one place to another. Helminths growth is promoted due to moisture in soil, because the ions which are needed for the development of eggs resides in soil, provide ability to eggs to hatch. Similarly, it was observed that 10 cm depth of moist soil was observed infested with STH eggs consistent with the observation of Rai et al.^33^. It was also observed that children were fond of to play in ponds and ingest infected eggs via faecal-oral route or through dirty unwashed hands. These observations are consistent with Vachel et al.^34^ in Philippines. Significant highest prevalence was observed in September and declined to the lowest in March due to decrease in temperature and humidity, unable eggs to hatch, therefore remain dormant in soil and seek their way to be transmitted to final host whenever chance is there. Stray or pet dogs and cats also defecate in the open areas, increases soil contamination, consistent with Mizgajska-Wiktor and Jarosz^35^.

The findings of the present study are less than observed 3.8% in Muzzafarabad^27^, 6.3% in Uganda^22^, 48. 8% in Brazil^23^,12.3% in Karachi^36^, 54.1% Nigeria^24^, 50.65% in Dhaka, Bangladesh^37^, 11.2% in China^14^, 16.5% in Zanzibar^38^, 53.6% in Brazil; 88.1% observed by Tarafder et al.^13,39^ and 22.8% in Gilgit, Pakistan^40^, 96.9% and 88.1%^41^ by using katokaz laboratory techniques. Parental education plays crucial role to provide good SES, health care facilities and better education in early childhood will return better outcomes in his late age. Especially mother education has great impact on socio-economic and demographic factors of households along with nutritional status of children. It was observed from the present study that parental education, father occupation, personal hygiene and social behavior of childlike pica have pronounced effects on child having ascariasis. These findings are supported by the results of Eraky et al.^42^. Health education is associated with GIP infection; it was observed in the present study that most of the parents especially mothers were uneducated and were ignorant of health-related measures. Maternal education plays significant role in promoting health of child. Many surveys have been conducted in different cities of Pakistan to observe the prevalence ratios of *A. lumbricoides* in soil samples contaminated by infected/carriers human or animal excreta. The previously performed research work by various researchers is not sufficient because it had been done in scanty form and data is scattered one. Similarly, it did not comprise the socioeconomic/demographic factors, i.e. 2.4% in Lahore^43^, 1.6% in Islamabad^44^, 4.1% in Islamabad and Larkana^45^, 1.0% in Muzzafarabad^27^, in Karachi^46^ and in Swat^47^ respectively. Various studies indicated that majority of the Parasite Intestinal Infections in secondary phase (PII) are asymptomatic^48^ and the carriers did not report to laboratories, as carriers are a serious threat for epidemics. Life span of adult worms in human intestine is about 2–7 years^49^. Colonoscopy was a useful diagnostic tool for trichiniasis^50^. Therefore, the present study was launched to cover all spectrum of prevalence to provide the researcher a milestone to do more, and helpful for the government sector to hit the affected areas to stop no more infestation/infection/disease by improving the SES.

A similar study conducted in Ethiopia also found significant differences in the prevalence of intestinal helminth infestation among six urban localities due to difference in SES^4^. The people of the studied areas have a change in conduct with more hygienic conducts to avoid helminths infection or disease. This was achieved by informing and consulting with the crowns, molvies and members of union councils and heads of families of the studied slums and the use of information banners or cards, visits of doctors or homoeopaths to free medical and homoeopathic camps installed for this purpose These observations are consistent with Alemu et al.^51^.

Parasitic infestation and diseases should be considered an important public health problem in Pakistan due to the association observed from various studies, among the prevailing SES, personal hygiene, infrastructural facilities and ascariasis. For the very first time in Pakistan present study has been designed based on SES to reveal distribution of *A. lumbricoide’*s eggs in naturally infected soil samples of urban areas of northern Lahore, to confirm inter relationship among used attributes like SES, inadequate sewage system, parental education, social and religious behavior of infected subjects. This study would be a milestone for health ministry and government to hit the affected areas for reduction of risk factors and beneficial to researchers. This study will provide a beneficial guideline to evaluate the prevailing risk factors in public health sectors in Pakistan.

## Conclusion

Unawareness about the biology of parasite, defecation in open fields promote soil contamination and infestation. Teaching programmes should be launched in schools, how to wash hands before taking food and after defecation. The present study could help the government to hit the infected areas; improve sanitation to reduce the level of transmission of ascariasis. Therefore, local executives should take strict implementation of local ordinances regulating the deworming programmes, exclusively start from schools to minimize the risk factors. As it is public health issue, government should have to include Hygiene and Physiology in academic course, also launch control and prevention strategies of this parasitosis. It is also crucial for teachers to emphasize to the children about the importance of washing hands especially after defecation and before eating anything. This data would be valuable to hit the specific areas of infection to cease further loss of manpower.
